# MoErv14 mediates the intracellular transport of cell membrane receptors to govern the appressorial formation and pathogenicity of *Magnaporthe oryzae*

**DOI:** 10.1371/journal.ppat.1011251

**Published:** 2023-04-03

**Authors:** Bin Qian, Xiaotong Su, Ziyuan Ye, Xinyu Liu, Muxing Liu, Haifeng Zhang, Ping Wang, Zhengguang Zhang

**Affiliations:** 1 Department of Plant Pathology, College of Plant Protection, Nanjing Agricultural University, and Key Laboratory of Integrated Management of Crop Diseases and Pests, Ministry of Education, Nanjing, China; 2 The Key Laboratory of Plant Immunity, Nanjing Agricultural University, Nanjing, China; 3 Department of Microbiology, Immunology, and Parasitology, Louisiana State University Health Sciences Center, New Orleans, Louisiana, United States of America; Institute of Microbiology, Chinese Academy of Sciences, CHINA

## Abstract

*Magnaporthe oryzae* causes rice blasts posing serious threats to food security worldwide. During infection, *M*. *oryzae* utilizes several transmembrane receptor proteins that sense cell surface cues to induce highly specialized infectious structures called appressoria. However, little is known about the mechanisms of intracellular receptor tracking and their function. Here, we described that disrupting the coat protein complex II (COPII) cargo protein MoErv14 severely affects appressorium formation and pathogenicity as the Δ*Moerv14* mutant is defective not only in cAMP production but also in the phosphorylation of the mitogen-activated protein kinase (MAPK) MoPmk1. Studies also showed that either externally supplementing cAMP or maintaining MoPmk1 phosphorylation suppresses the observed defects in the Δ*Moerv14* strain. Importantly, MoErv14 is found to regulate the transport of MoPth11, a membrane receptor functioning upstream of G-protein/cAMP signaling, and MoWish and MoSho1 function upstream of the Pmk1-MAPK pathway. In summary, our studies elucidate the mechanism by which the COPII protein MoErv14 plays an important function in regulating the transport of receptors involved in the appressorium formation and virulence of the blast fungus.

## Author summary

During the pathogenic interaction with rice, *Magnaporthe oryzae* senses cell surface cues and forms a highly specialized infectious structure called an appressorium that initiates the infection. Studies have shown several transmembrane proteins essential for the perception of rice host cues. However, little is known about how these transmembrane proteins are trafficked intracellularly to impact appressorium formation and pathogenicity. In this study, we found MoErv14 is important in the growth and pathogenicity of the blast fungus, and MoErv14 regulates the transport of several membrane sensors, including MoPth11, MoWish, and MoSho1, that function upstream of either G-protein/cAMP signaling or the Pmk1-MAPK pathway. Importantly, we revealed that MoErv14 mediates the transport of these receptor proteins to promote the appressorium formation and pathogenicity of *M*. *oryzae*.

## Introduction

Rice blast is one of the most devastating fungal diseases on rice, and it seriously threats world food supply security [1]. When infecting the host, the blast fungus *Magnaporthe oryzae* recognizes external physical and chemical signals, such as surface hardness and hydrophobicity, to produce specific infectious structures called appressoria. The robust turgor generated in appressoria leads to the penetration of the host epidermis and the production of invasive hyphae.

Previous studies indicated that several cell surface transmembrane receptor proteins, including MoPth11, play a role in sensing the host surface for appressorium production. MoPth11 is an atypical G protein-coupled receptor (GPCR) that senses surface hydrophobicity leading to endocytosis, which regulates the cAMP-signal pathway and appressorium formation [2,3]. In addition, the receptor proteins MoMsb2 and MoSho1 recognize the host surface keratinous monomer to activate the MoMst11-MoMst7-MoPmk1 MAP kinase signaling pathway that also controls appressorium formation. A recent study suggested that a GPCR protein, MoWish, is involved in appressorium formation by also recognizing host hydrophobic signals [4]. Although the above studies showed that surface transmembrane proteins are important for signal perception and appressorium induction, the molecular mechanism by which these transmembrane proteins are transported intracellularly and exert their function remains unknown.

In eukaryotic cells, protein transport and exchange of macromolecular substances between different organelles depend on vesicle trafficking. Vesicles are membrane-coated cargos containing various macromolecules that shuttle between different subcellular structures or organelles. According to their different forms of membrane coating, vesicles are divided into clathrin vesicles, COPI (cytoplasmic envelope complex I or coat protein complex I) vesicles, and COPII (cytoplasmic envelope complex II, coat protein complex II) vesicles. Clathrin vesicles mainly function in the transport of cargo from the Golgi apparatus to lysosomes and cell membranes and the endocytosis of extracellular or membrane substances into the cell. COPI vesicles mediate transport from the Golgi back to the endoplasmic reticulum (ER), and COPII vesicles mediate the material transport from the ER to the Golgi [5].

The transport of substances from the ER to the Golgi apparatus mediated by COPII is an essential component of the secretion pathway in eukaryotes that is essential for cells to perform their normal functions and maintain the dynamic balance of various organelles [6]. For example, in the budding yeast *Saccharomyces cerevisiae*, the Sec23/Sec24 complex forms the inner membrane, while the Sec13/Sec31 complex forms the outer membrane when coating on COPII vesicles [7]. ERV (ER-derived vesicles) proteins, divided according to their molecular weights [8,9], are a group of conserved cargo receptor proteins that is mainly responsible for recruiting specific cargo proteins and directing their ER to Golgi transport [9]. Different ERV proteins recognize specific cargo proteins [10], but the transport of the cargo containing transmembrane receptor proteins remains unclear.

We previously reported that MoErv29 specifically mediates the secretion of protein effectors during infection [11]. Here, we characterized the function of MoErv14, another member of the COPII vesicle receptor family in *M*. *oryzae*, and we revealed that MoErv14 mediates the transport of various transmembrane receptor proteins that promote the appressorium formation and pathogenicity of *M*. *oryzae*.

## Results

### Identification and characterization by host infection of ERV family genes in *M*. *oryzae*

**Fig 1:**
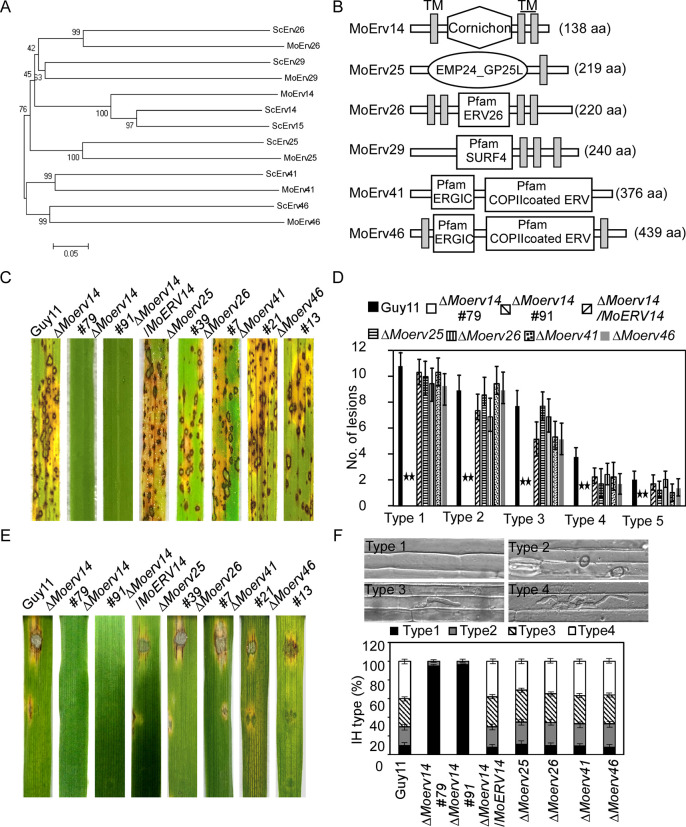
Prediction, phylogenetic analysis, and virulence of ERV (ER-derived vesicles) proteins in *M*. *oryzae*. (A) Phylogenetic trees of ERV proteins from several organisms were constructed using the CLUSTAL_W, and MEGA 5.1 programs by the neighbor-joining method with 1000 bootstrap replicates. (B) Schematic representation of ERV proteins in *M*. *oryzae*. (C) Two-week-old rice seedlings (*Oryza sativa* cv. CO-39) were sprayed with tested with a conidial suspension of different mutants (5×10^4^ conidia/ml). The diseased leaves were harvested and photographed 7 days after inoculation. Three independent experiments were performed. (D) Quantification of lesion types (per 1.5 cm^2^) on susceptible rice leaves. 0, no lesion; 1, dark-brown pinpoint lesions; 2, 1.5 mm brown spots; 3, 2–3 mm lesions with brown margins; 4, eyespot lesions longer than 3 mm; 5, coalesced lesions infecting 50% or more of the leaf maximum size. Asterisks represent significant differences (*p*<0.01). (E) Separated barley leaves were dropped with serial dilution (1×10^5^, 1×10^4^, 1×10^3^ conidia /ml) of conidial suspensions, and diseased leaves were photographed 5 days after inoculation. (F) Conidial suspensions (1×10^5^ spores/ml) of three different strains were injected into separate rice sheaths, and the infection severity was observed at 24 h post-inoculation (hpi). Percentages of different types of infectious hyphae in rice cells were counted at 24 hpi. Sample size (n) = 50. Error bars represent the standard deviations. Type 1, no infection structures; Type 2, only with a appressorium; Type 3, only with a single invasive hypha (IH); Type 4, with 1–3 branches but restricted in one cell.

We previously reported that the COPII cargo receptor MoErv29 promotes the apoplastic effector secretion and the virulence of *M*. *oryzae* [11]. In order to systematically elucidate functions of the ERV COPII cargo receptor family proteins, we screened the available genomes of *M*. *oryzae* by BLAST using the *S*. *cerevisiae* ERV proteins’ sequence as a reference and identified five *MoERV* genes in *M*. *oryzae*: *MoERV14* (MGG_08132), *MoERV25* (MGG_08210), *MoERV26* (MGG_08706), *MoERV41* (MGG_00949), and *MoERV46* (MGG_01245) (Fig 1A and 1B).

To examine the roles of the above-identified ERV proteins in *M*. *oryzae*, we generated respective mutant strains that were verified by Southern blot analysis (S1 Fig) and carried out pathogenicity studies. *C*onidial suspensions of the mutants were sprayed onto 2-week-old rice seedlings (*Oryza sativa* cv. CO-39), along with the wild-type strain Guy11. Seven days after inoculation, Δ*Moerv14* mutants failed to produce any leaf lesions, while Δ*Moerv25*, Δ*Moerv26*, Δ*Moerv41*, and Δ*Moerv46* all produced numerous spindle-shaped lesions, comparable to Guy11. Similar results were also observed on detached barley leaves. However, in the rice-sheath penetration assay, lesions produced by the Δ*Moerv14* mutant showed 95% Type 1 and 5% Type 2 infectious hyphal growth, compared to 9% Type 1, 20% Type 2, 31% Type 3, and 40% Type 4 in Guy11. The severe defect in the pathogenicity of Δ*Moerv14* was restored in the complemented strain (Fig 1C–1F). We concluded that MoErv14 plays a critical role in host penetration and invasive hyphae growth.

### MoErv14 is involved in the vegetative growth, conidium formation, and appressorium development of *M*. *oryzae*

**Fig 2:**
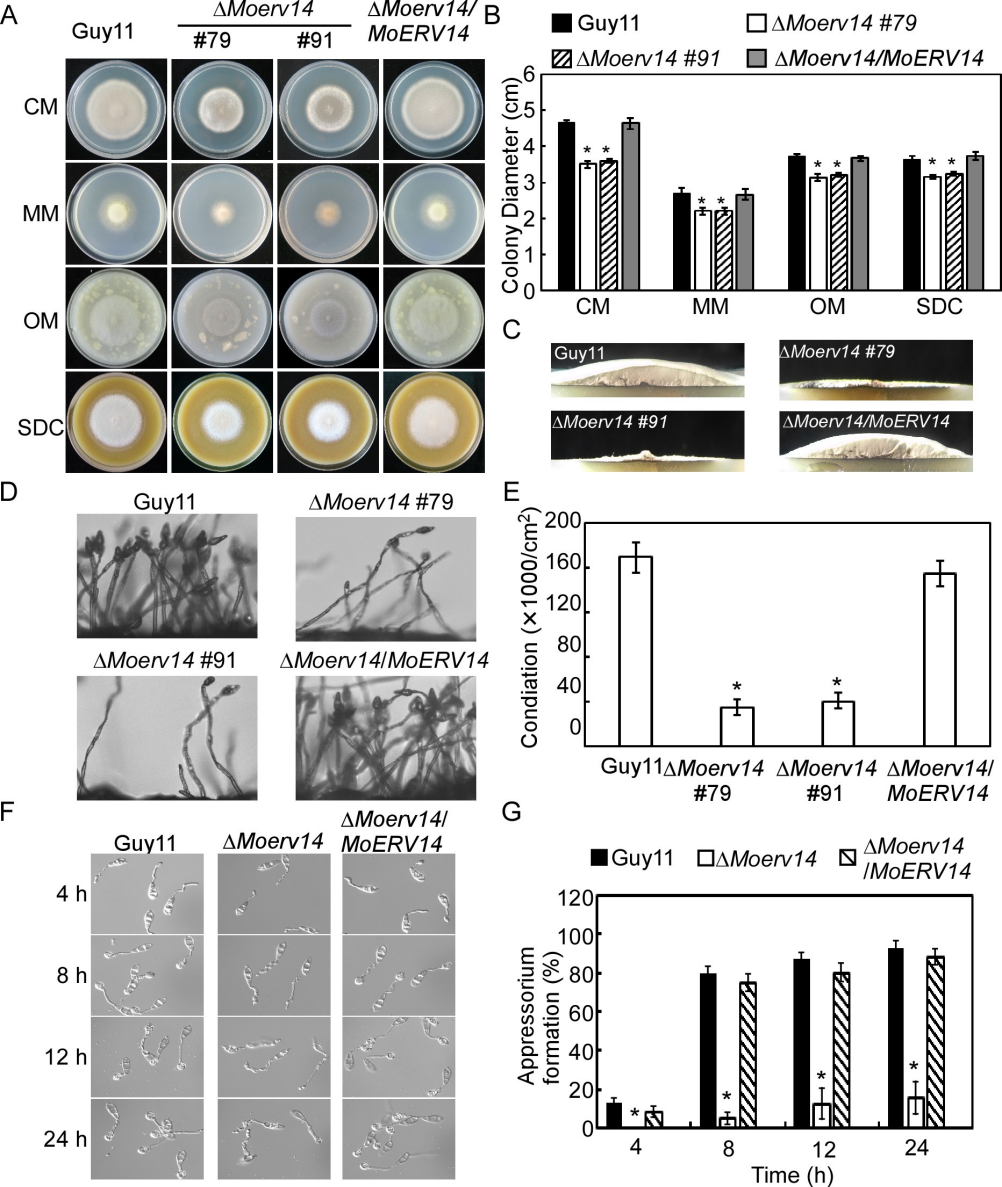
MoErv14 is involved in the vegetative growth, conidium formation, and appressorium development. (A) The tested strains Guy11, Δ*Moerv14* mutants, and complemented strain were inoculated on CM, MM, OM, and SDC media, 28°C for 7 days, and then photographed. (B) Statistical analysis of the colony diameter of wild-type Guy11, Δ*Moerv14* mutants and complemented strain on different media. Error bars represent the standard deviations; Asterisks denote statistical significances (*p*<0.01). (C) The Δ*Moerv14* mutant appears as a flat colony with thin aerial hyphal growth when compared with the wild type Guy11. (D) Conidia of different strains were observed under a light microscope after illumination for 24 h and then photographed. (E) The number of conidia was calculated and analyzed from the tested strains following incubation on SDC medium for 7 days. Error bars represent the standard deviations. Asterisks represent a significant difference (*p*<0.01). (F) A comparative time-lapse observation about the conidia germination and appressorium formation of the Δ*Moerv14* mutant and the wild type Guy11 at 4, 8, 12, and 24 h time points. (G) Statistical analysis of the appressorium formation observed at 4, 8, 12 and 24 h time point. Error bars represent the standard deviations. Asterisks represent significant differences. (*p*<0.01).

We selected MoErv14 for further studies based on its importance in pathogenicity. Firstly, to determine whether Erv14 is conserved among species, we used cDNA of MoErv14 to complement a yeast Δ*erv14* mutant. As expected, MoErv14 could partially suppress the growth defect of the yeast Δ*erv14* mutant upon dithiothreitol (DTT)-induced stress (S2 Fig). Despite the partial suppression, it suggested that MoErv14 could be functionally homologous to ScErv14. We then observed the vegetative growth of Δ*Moerv14* mutants that showed significant growth reduction on CM, MM, SDC, and OM media in comparison to Guy11 and the complemented strain (Fig 2A–2B). The colony formed by the Δ*Moerv14* mutant was much thinner on day 7 (Fig 2C). We also found that Δ*Moerv14* mutants produced significantly fewer conidia on cornmeal agar (SDC) (Fig 2D–2E). These results indicated that MoErv14 is important for the hyphae growth and asexual development of *M*. *oryzae*.

To further assess hyphae growth and asexual development as the cause of decreased virulence of the Δ*Moerv14* mutant, we estimated conidia germination and appressorium formation at various time points [12–14]. The conidia germination of the Δ*Moerv14* mutant was not significantly different from Guy11. However, its appressorium formation rate was significantly lower than Guy11, as ~20% of the conidia developed appressoria in 24 hours (Fig 2F–2G). Meanwhile, the appressoria of the Δ*Moerv14* mutant showed a higher collapse ratio than the control, indicating a decreased turgor pressure (S3 Fig). In addition, MoErv14 appeared not to be involved in a septin ring formation (S4 Fig). This results suggested that MoErv14 is important for appressorium formation and function.

### MoErv14 is a vesicle traffic-associated protein

**Fig 3:**
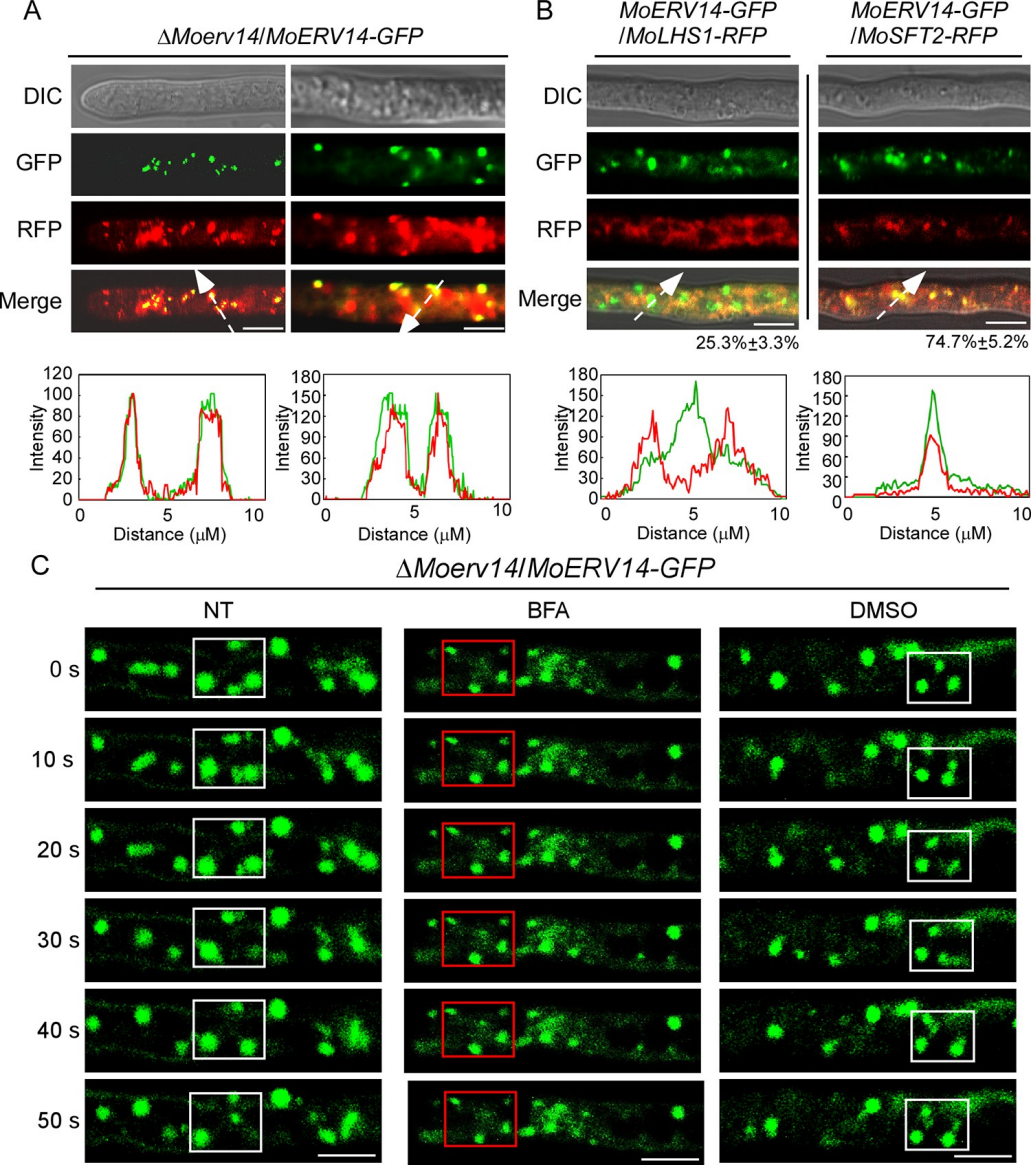
MoErv14 shuttles between ER to Golgi in *M*. *oryzae*. (A) MoErv14-GFP were expressed in Δ*Moerv14*, then stained with FM4-64 for 3min and observed by Axio Observer A1 Zeiss inverted microscope. The left shown is the tip of the hypha and the right shown is the middle of the hyphae. Asterisks represent the labeled and merged vesicles. Bar = 10 nm. (B) The localization pattern of MoErv14 in the hypha. Shown are confocal fluorescent images (Zeiss LSM710, 63×oil). Bar = 10 nm. (C) Time-lapse images of cells expressing the MoErv14-GFP reporter at different time intervals with or without the treatment of ER-Golgi protein trafficking inhibitor Brefeldin A (BFA), 50 μg/ml, DMSO treatment was used as a control, Bar = 10 nm. White frames indicated dynamic MoErv14 and red frames indicated detained MoErv14.

We then generated a MoErv14-GFP fusion protein and found MoErv14-GFP is located at vesicle-like spots that could be stained by the lipophilic dye FM4-64 (Fig 3A). With reference to RFP-tagged MoLhs1 and MoSft2 proteins that are markers of ER and Golgi, respectively [15–17], we observed about 25% of MoErv14-GFP co-localized or was surrounded by MoLhs1, and there was nearly 75% MoErv14-GFP co-localized with MoSft2 (Fig 3B). Similar results were also observed during conidia and appressorium stages in that about ~80% MoErv14 localized in Golgi and ~20% MoErv14 in ER, respectively (S5A and S5B Fig).

Moreover, by using gradient centrifugation to fractionate subcellular organelles, we confirmed that MoErv14 was mainly accumulated in Golgi and ER (S5C Fig). We further co-expressed MoErv14-S and MoSec24-2-GFP in Guy11 and extracted the vesicular proteins. Proteins were then analyzed by Western blotting using S tag and GFP-specific antibodies. The results showed that MoErv14 and MoSec24-2 were detected in total vesicular proteins, which indicated that MoErv14 is a vesicular protein (S5D Fig). In yeast, Erv14 was reported to function as a COPII component shuttling between ER and Golgi. We then employed Brefeldin A (BFA, an inhibitor of ER to Golgi protein transport) and live-cell time-lapse imaging analysis revealed that the dynamic localization pattern of MoErv14 can be specifically inhibited by BFA (Fig 3C) [18]. Taken together, these results suggested that MoErv14 is a COPII component that shuttles between ER and Golgi.

We also examined whether the Δ*Moerv14* mutant is defective in endocytosis. By staining cells with FM4-64, we observed its internalization, but there was no apparent difference between the Δ*Moerv14* mutant and Guy11 2 and 5 min after staining (S6 Fig).

### MoErv14 mediates cAMP and MoPmk1-MAPK signaling pathways

Previous studies demonstrate that both cAMP and MoPmk1-MAPK signaling pathways are implicated in transducing hydrophobic surface signals to regulate appressorium formation in *M*. *oryzae* [1]. When MoPth11 senses surface signals, cAMP signaling is activated to promote appressorium formation. MoMsb2 and MoSho1, on the other hand, control the formation of appressoria by sensing host surface signals to activate the Mst11-Mst7-Pmk1 signaling pathway through protein phosphorylation. We measured cellular levels of cAMP and MoPmk1 phosphorylation, respectively, and the results showed that both cAMP levels and MoPmk1 phosphorylation were decreased significantly in the Δ*Moerv14* mutant (S7A–S7B Fig).

**Fig 4:**
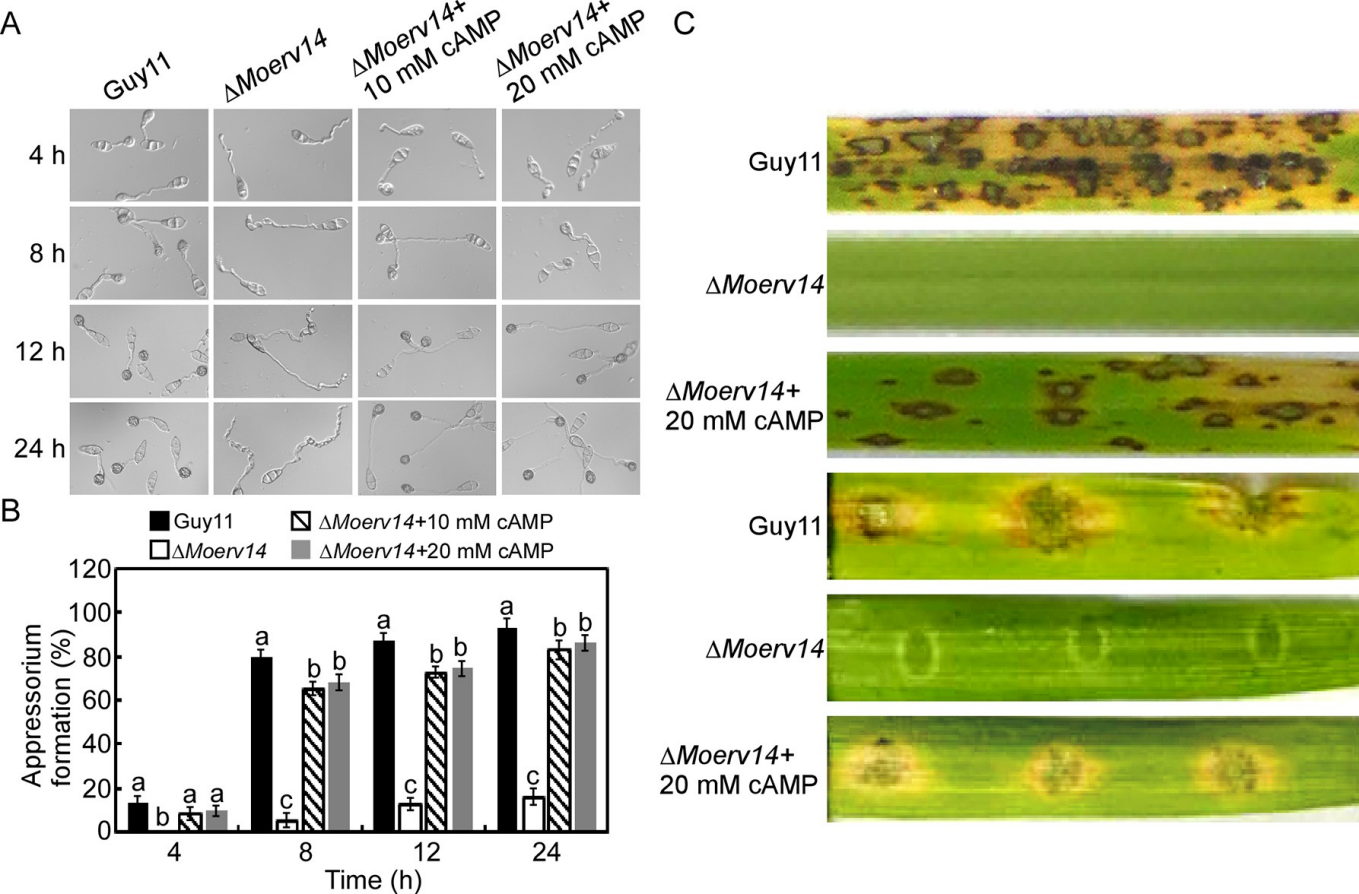
MoErv14 is important for the appressorium formation and virulence by regulating cAMP level in *M*. *oryzae*. (A) Exogenous cAMP suppressed the appressorium formation defect of the Δ*Moerv14* mutant. (B) Statistical analysis of the appressorium formation rate. Error bars represent ±standard deviations (SD), and different numbers denote statistical significances (*p*< 0.01). (C) Two-week-old rice seedlings (*Oryza sativa* cv. CO-39) were sprayed with tested with a conidial suspension of different mutants (5×10^4^ conidia/ml). The diseased leaves were harvested and photographed 7 days after inoculation. Three independent experiments were performed. Separated barley leaves were dropped with serial dilution (5×10^4^ conidia /ml) of conidial suspensions, and diseased leaves were photographed 5 days after inoculation.

We further analyzed the effect of externally supplementing cAMP on the Δ*Moerv14* mutant. At 10 and 20 mM levels, the Δ*Moerv14* mutant can germinate and form appressoria, but not at the levels of the wild-type strain (Fig 4A–4B). Exogenous cAMP also suppressed the virulence defect of Δ*Moerv14* mutants on rice and barley (Fig 4C).

**Fig 5:**
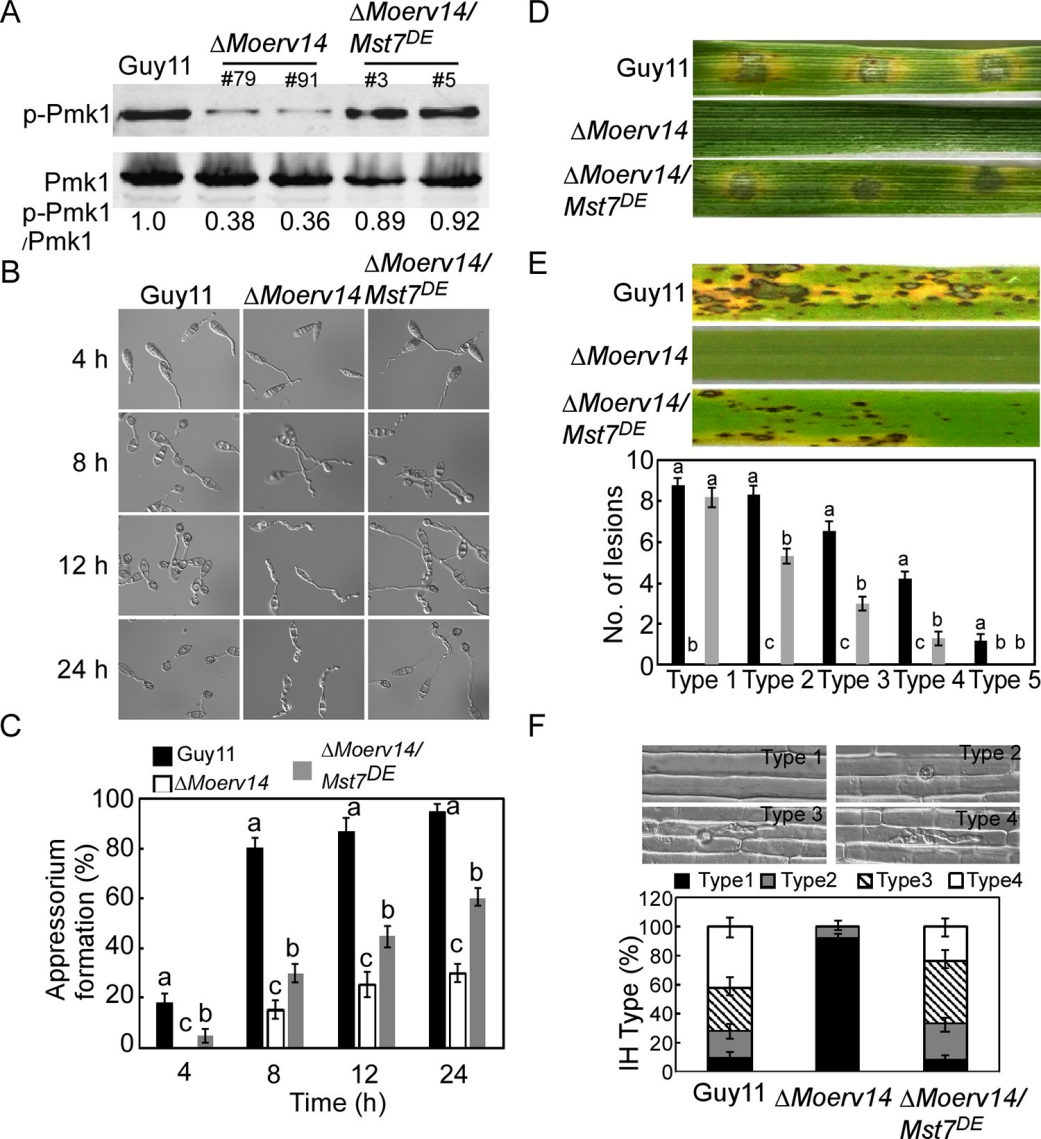
Activating the Pmk1-MAPK signaling pathway suppresses the defect of appressorium formation in the Δ*Moerv14* mutant. (A) Pmk1 phosphorylation level analysis with proteins extracted from the mycelia of Guy11, Δ*Moerv14* mutant, and Δ*Moerv14/Mst7*^*DE*^. The phosphorylation levels of Pmk1 (42-kDa) were detected using a phosphor-MAPK antibody (upper panel). The endogenous Pmk1 was detected using a MAPK antibody (lower panel). (B) Appressorium formation assay on the hydrophobic surfaces at 4 h, 8 h, 12 h and 24 h, respectively. (C) Appressorium formation rates were calculated and statistically analyzed. Asterisks represent significant differences (*p<0*.*01*). (D) Pathogenicity assay on detached barley leaves. (E) Pathogenicity assay on rice (CO-39) and the quantification of the lesion numbers per 5 cm length of rice leaf. Error bars represent SD and different numbers denote statistical significances (*p*< 0.01). (F) Penetration assays in rice sheath. IH growth on rice cells was observed at 24 hpi and 4 types of IH were quantified and statistically analyzed. Error bars represent SD. Asterisks represent expanded IH.

We also transformed a *MoMST7*^S212D, T216E^ (*MoMST7*^*DE*^) construct into the **Δ***Moerv14* mutant to constitutively activate the MoPmk1-MAPK1 pathway (Fig 5A). The results showed that MoMst7^DE^ effectively suppressed the defect of the **Δ***Moerv14* mutant in appressorium formation (Fig 5B–5C). Seven days after inoculation, the virulence of Δ*Moerv14*/*MoMST7*^*DE*^ was partially restored when compared with the Δ*Moerv14* mutant. Δ*Moerv14*/*MoMST7*^*DE*^ also exhibited a higher penetration rate and enhanced infectious hyphal growth (Fig 5D–5F). Based on these results, we concluded that MoErv14 is involved in regulating cAMP and MoPmk1-MAPK signaling pathways required for appressorium formation and pathogenicity.

### MoErv14 interacts with and mediates the transport of MoPth11, MoWish, and MoSho1

**Fig 6:**
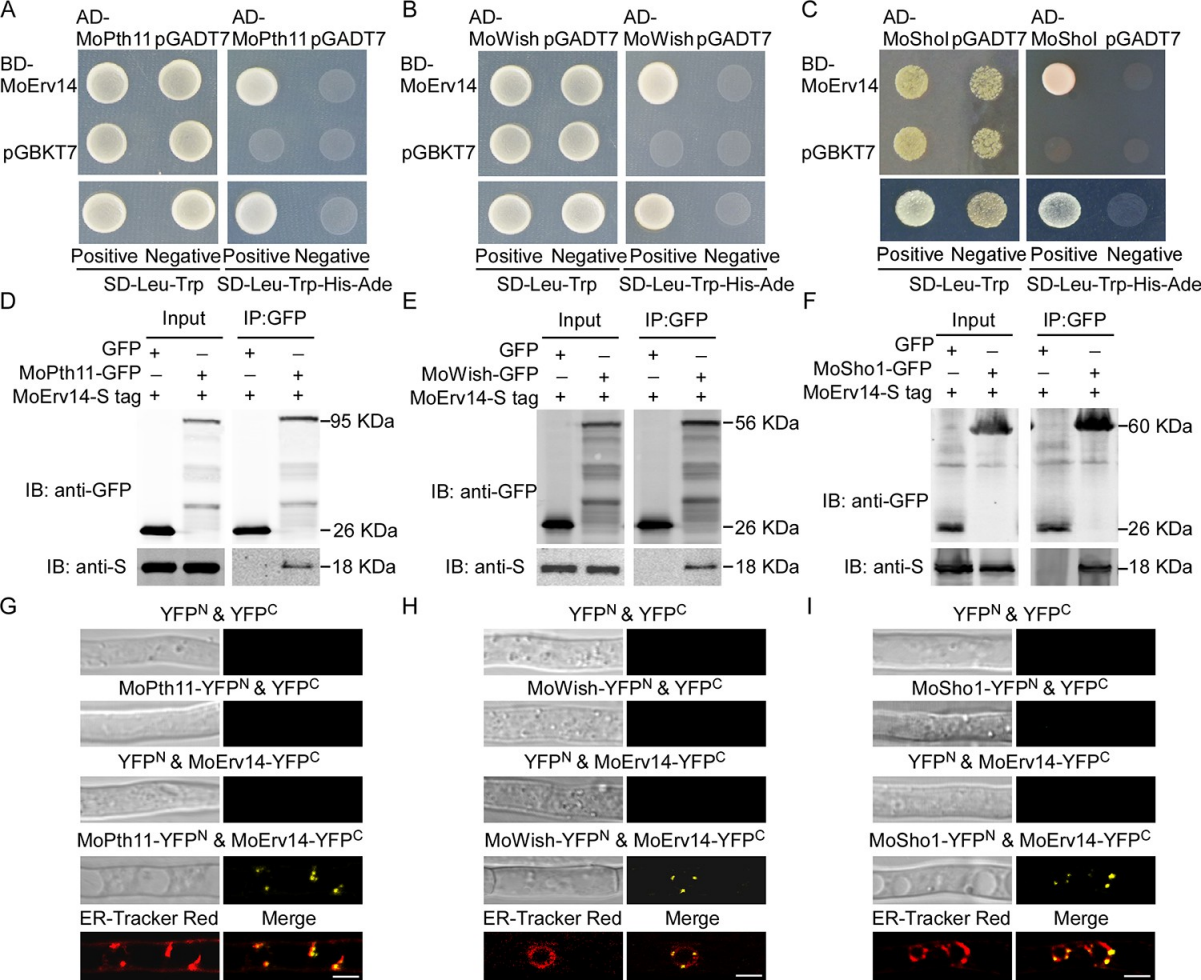
MoErv14 interacts with MoPth11, MoWish, and MoSho1. (A-C) Yeast two-hybrid assay for interactions of MoErv14 with MoPth11, MoWish, and MoSho1. (D-F) Co-immunoprecipitation (Co-IP) assays to detect interactions of MoErv14 with MoPth11, MoWish, and MoSho1. Total proteins were extracted from MoErv14-S tag/MoPth11-GFP, MoErv14-S tag/MoWish-GFP, MoErv14-S tag/MoWish-GFP co-expressing strains, and the MoErv14-S tag/GFP-empty strain. Proteins were then incubated with anti-GFP agarose before being eluted and detected with anti-S tag and anti-GFP antibodies, respectively. (G-I) BiFC assay for the MoPth11 & MoErv14, MoWish & MoErv14 and MoSho1 & MoErv14 interaction in vivo. Hyphae were treated with BFA and examined by DIC and fluorescence microscopy. The strains expressing the MoPth11-YFP^N^ & YFP^C^, MoErv14-YFP^C^ & empty YFP^N^, MoWish-YFP^N^ & YFP^C^, MoSho1-YFP^N^ & YFP^C^, and empty YFP^N^ and empty YFP^C^ constructs were used as controls. Dye ER-Tracker Red was used to image ER in the interacting strains and merged. Bars = 10 μm.

Previous studies suggested that transmembrane receptors, including MoPth11, MoWish, and MoSho1, are important for cell surface perception and appressorium formation in *M*. *oryzae* [3,4,19]. We demonstrated that MoErv14 regulates cAMP and MoPmk1-MAPK signaling pathways during appressorium formation and pathogenicity. To test whether MoErv14 interacts with these receptors, yeast two-hybrid (Y2H) and co-immunoprecipitation (co-IP) assays were conducted that showed MoErv14 interacting with all three proteins (Fig 6A–6F). Also, bimolecular fluorescence complementation (BiFC) was used to confirm the interaction between MoErv14 and these proteins following BFA treatment (Fig 6G–6I). However, no interaction was detected among these transmembrane receptor proteins (S8A–S8C Fig).

**Fig 7:**
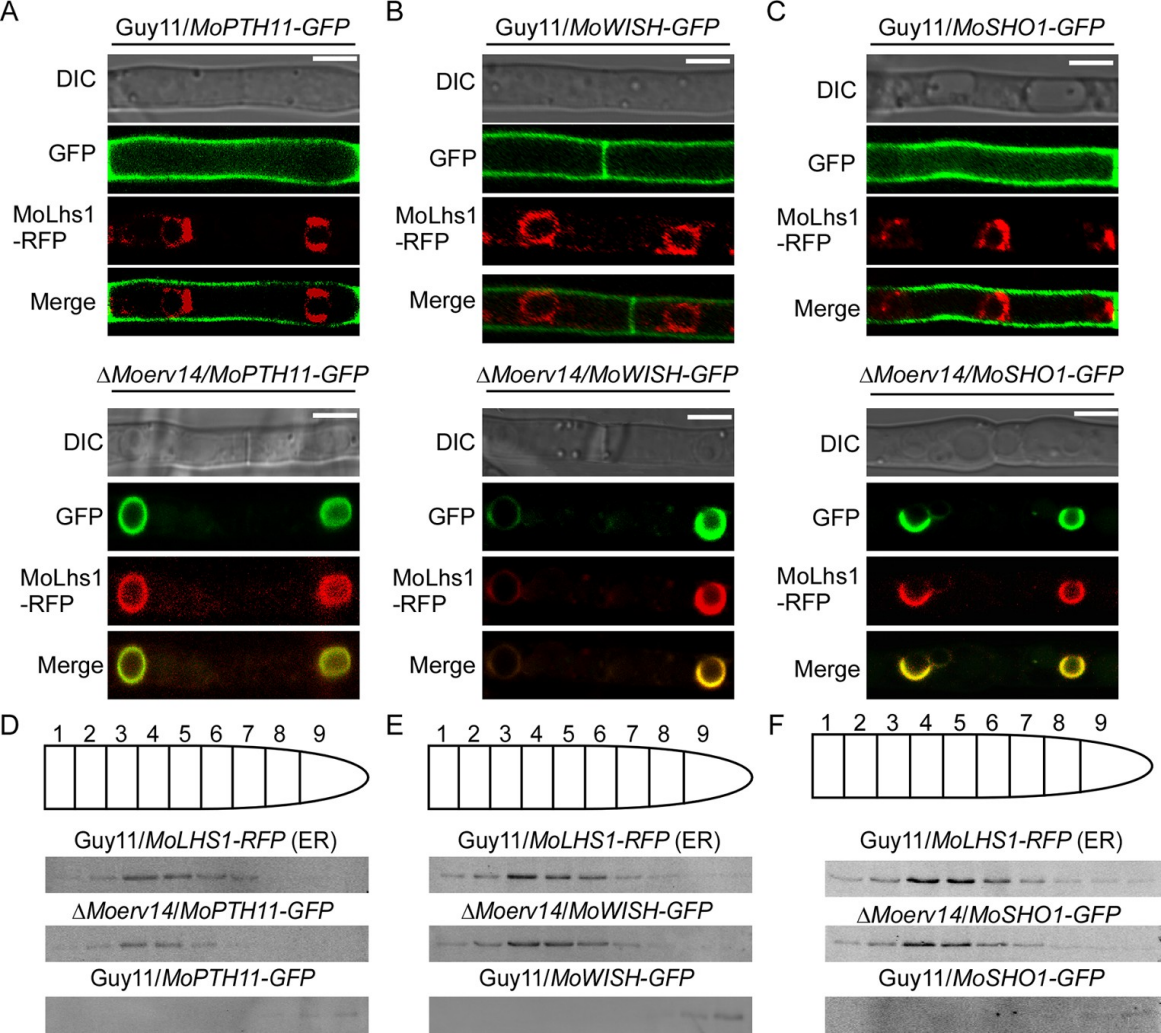
MoErv14 is involved in the transport of MoPth11, MoWish, and MoSho1. (A-C) The subcellular localization of MoPth11-GFP, MoWish-GFP, and MoSho1-GFP in Guy11 and the Δ*Moer*v14 mutant during hyphal stage. MoPth11-GFP, MoWish-GFP, and MoSho1-GFP were localized in the cell membrane of the wild-type and in ER of the Δ*Moerv14* mutant. Bars, 10 μm. (D-F) Organelles from *M*. *oryzae* protoplasts were partially separated by centrifugation. The ER distribution and gradient fractions were analyzed by Western blotting using RFP antibodies against the ER marker MoLhs1 fused with RFP. Distribution of MoPth11 was detected by the anti-GFP antibody.

As MoErv14 functions in COPII vesicle trafficking, we speculated that MoErv14 also interacts with these proteins to control their transport. Hence, we fused GFP with these proteins and expressed them in Guy11 and the Δ*Moerv14* mutant, respectively. The results showed that MoPth11, MoWish, and MoSho1 were all located at the cell membrane surface of Guy11. However, in Δ*Moerv14* mutants, they were all retained in ER (Fig 7A–7C), which was similar with the BFA treatment of Guy11 (S9 Fig). Using gradient centrifugation to fractionate subcellular organelles, we found that MoPth11-GFP, MoWish-GFP, and MoSho1-GFP were mainly distributed in ER fractions of the Δ*Moerv14* mutant, along with MoLhs1 (Fig 7D–7F).

**Fig 8:**
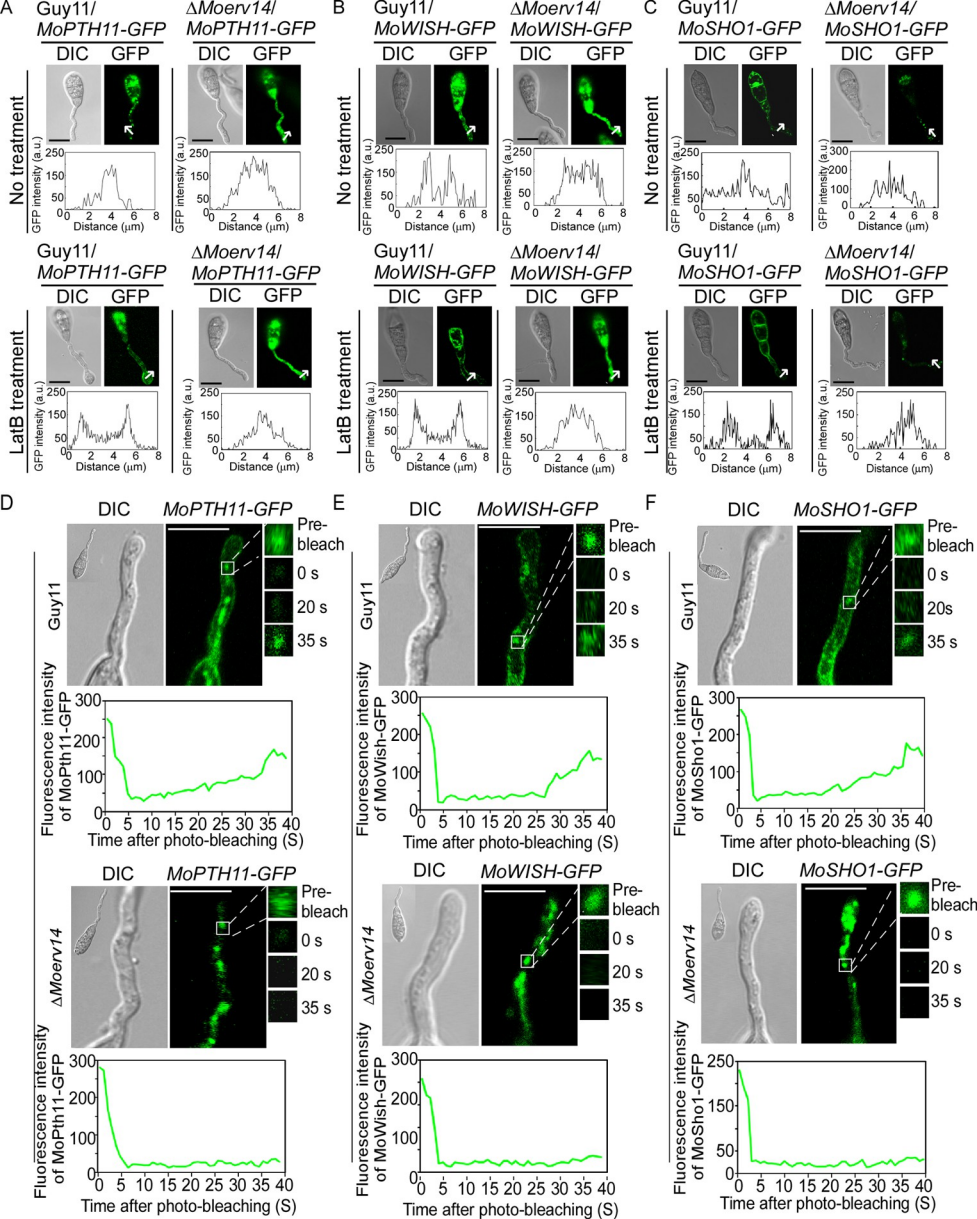
MoErv14 regulates trafficking of MoPth11-GFP, MoWish-GFP, and MoSho1-GFP during conidial germination. (A) MoPth11-GFP localization patterns in germ tubes of Guy11 and Δ*Moerv14* mutant at 3 h and MoPth11-GFP localization patterns with LatB treatment in germ tubes of Guy11 at 3 h. Bars = 10 μm. (B) MoWish-GFP localization patterns in germ tubes of Guy11 and Δ*Moerv14* mutant at 3 h. MoWish-GFP localization patterns with LatB treatment in germ tubes of Guy11 at 3 h. Bars = 10 μm. (C) MoSho1-GFP localization patterns in germ tubes of Guy11 and Δ*Moerv14* mutant at 3 h. MoSho1-GFP localization patterns with LatB treatment in germ tubes of Guy11 at 3 h. Bars = 10 μm. (D) Representative images of FRAP analysis for diffusion at MoPth11-GFP localized regions in germ tubes of Guy11 and Δ*Moerv14*. The fluorescence of MoPth11-GFP significantly recovered at 35 s post-photobleaching in Guy11 but not in Δ*Moerv14*. The FRAP curves of MoPth11-GFP localized regions in Guy11 and Δ*Moerv14*. 20 regions from different cells were subjected to FRAP analysis for each strain. Intervals: 5 s. Bars = 5 μm. (E) Representative images of FRAP analysis for diffusion at MoWish-GFP localized regions in germ tubes of Guy11 and Δ*Moerv14*. The fluorescence of MoWish-GFP significantly recovered at 35 s post-photobleaching in Guy11 but not in Δ*Moerv14*. The FRAP curves of MoWish-GFP localized regions in Guy11 and Δ*Moerv14*. 20 regions from different cells were subjected to FRAP analysis for each strain. Intervals: 5 s. Bars = 5 μm. (F) Representative images of FRAP analysis for diffusion at MoSho1-GFP localized regions in germ tubes of Guy11 and Δ*Moerv14*. The fluorescence of MoSho1-GFP significantly recovered at 35 s post-photobleaching in Guy11 but not in Δ*Moerv14*. The FRAP curves of MoSho1-GFP localized regions in Guy11 and Δ*Moerv14*. 20 regions from different cells were subjected to FRAP analysis for each strain. Intervals: 5 s. Bars = 5 μm.

To distinguish whether these proteins were transported to the cell surface, we observed their locations with or without Latrunculin B (LatB) which inhibits endocytosis. In Guy11 and during the appressorium formation stage, these proteins were transported into the cytoplasm by endocytosis from the cell membrane following signal perception to mediate appressorial formation. In the presence of LatB, these proteins were retained in the cell membrane surface. In contrast, these proteins failed in transport to the cell surface and were trapped on the ER in the mutants, with or without LatB treatment (Fig 8A–8C). We further compared the Δ*Moerv14* mutant and Guy11 in the transport of MoPth11, MoWish, and MoSho1 by fluorescence recovery after photobleaching (FRAP). We intended to bleach the fluorescence from the regions where MoPth11-GFP, MoWish-GFP, and MoSho1-GFP were accumulated in germ tubes, and the recovery of fluorescence can reflect the rate of endocytosis. In the FRAP assay, we bleached 90% of fluorescence of a region using 488 nm light. For MoPth11-GFP, fluorescence was recovered at post-photobleach 35 s in Guy11, but not in the Δ*Moerv14* mutant. In addition, the recovery level of MoWish-GFP and MoSho1-GFP in the Δ*Moerv14* mutant was also significantly impaired when compared with Guy11 at the same post-photobleach (Fig 8D–8F). Collectively, these results suggested that MoErv14 is involved in the transport of MoPth11, MoWish, and MoSho1 to the Golgi, and MoErv14 affects the transportation of these proteins to the cell membrane.

## Discussion

**Fig 9:**
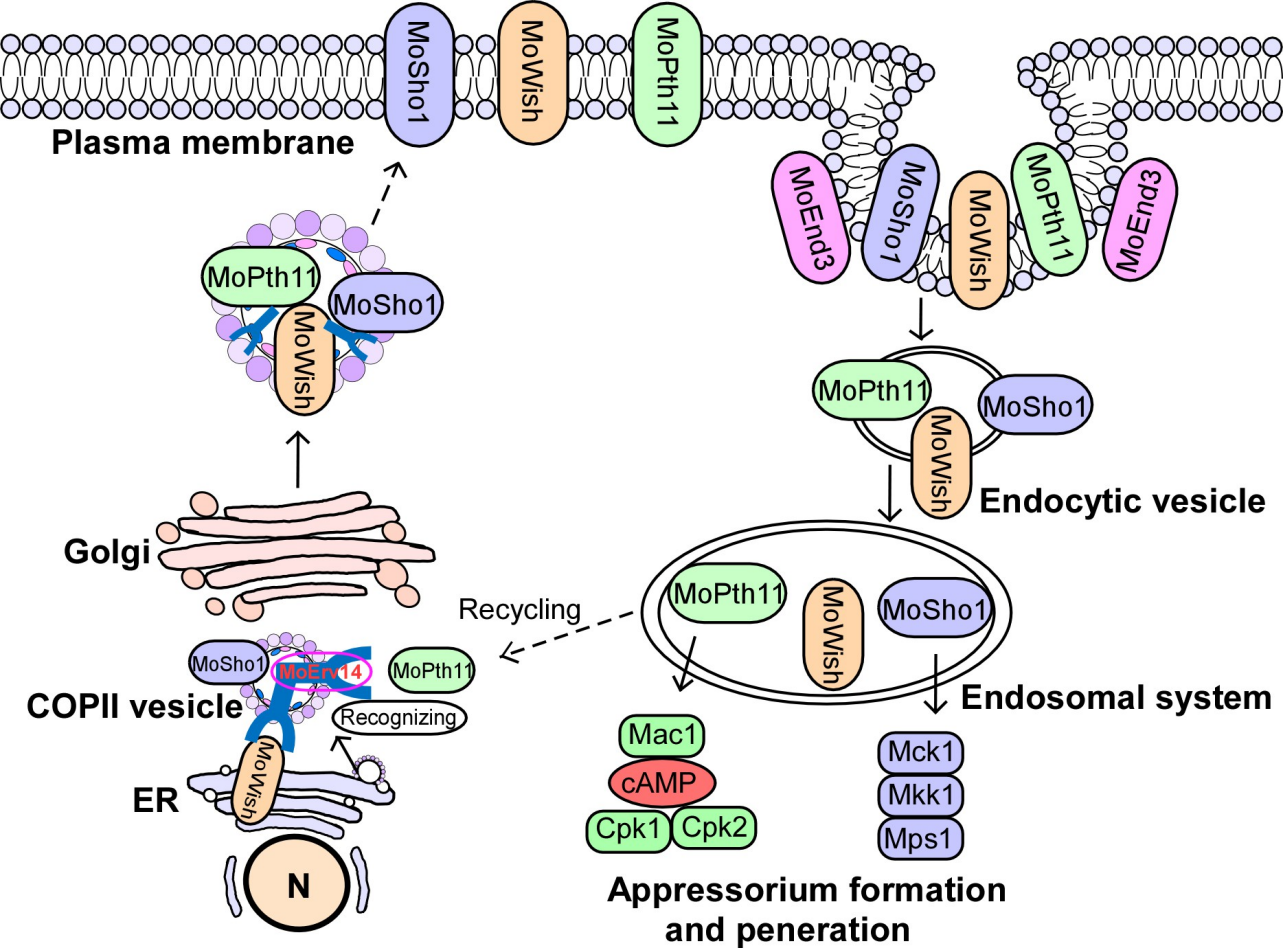
A proposed working model of MoErv14 function. During germ tube development, GPCR Pth11, Wish, and membrane sensor MoSho1 are recognized and transported by MoErv14. After seining the plant surface signals the membrane sensors are regulated by MoEnd3-mediated endocytosis. Following transport to endosomal systems by endocytic vesicles, MoPth11, MoWish, and MoSho1 can trigger a downstream Pmk1-MAPK and CWI MAPK cascade. The MAPK cascade facilitates successful appressorium formation, penetration, and pathogenicity.

Understanding the mechanisms by which pathogen transmembrane receptors transduce plant surface signals promotes the development of strategies in controlling the rice blast. Previous studies revealed that MoEnd3 and MoCrn1 undergo endocytosis following the perception of external signals in *M*. *oryzae* [20,21]. This current study demonstrated that MoErv14 mediated the signal transduction of receptors MoPth11 and MoSho1 by governing their intracellular transport. MoPth11 and MoSho1 regulate cAMP and Pmk1-MAPK signaling pathways, respectively, to promote appressorium formation and pathogenicity of the blast fungus. Based on these results, we concluded that COPII coat complex proteins, such as MoErv14, played critical roles in recognizing and transmitting host surface signals (Fig 9).

Previous studies also showed that COPII coat complex proteins-mediated ER to Golgi transport is an important component of eukaryotic secretion pathways [22]. In *S*. *cerevisiae*, individual ERV proteins recognize the different groups of cargo proteins and are important in ER-Golgi transport [8,23–25]. In *M*. *oryzae*, this process is closely linked to its physiology and pathogenicity. Following our previous studies of MoErv29 promoting apoplastic effector secretion contributing to the virulence of *M*. *oryzae* [11], we here showed that another ERV protein, MoErv14, was involved in appressorium formation and pathogenicity by governing intracellular transport of several receptor proteins. In addition, even though we found a severe defect of appressorium formation in the Δ*Moerv14* mutant, no defect was found in conidium germination. Similar results were also observed in the Δ*Mowish* and Δ*Mosho1* mutants [4,19]. These observations indicated that conidium germination may be regulated by different signal pathways.

The increased knowledge of each specific cargo receptor makes it feasible to hypothesize the presence of specificities between cargo coat proteins and cargo contents. Indeed, the examination of the cargo spectrum relying on ScErv14 suggested that this large and non-homogenous group of proteins is recognized based on the length of their transmembrane domains (TMDs), rather than their sequences [26]. We have noticed that MoPth11, MoSho1, and MoWish all contain a long TMD; however, MoErv14 is not known to regulate MoMsb2 that also contains a long TMD. These findings indicated that the mechanisms of MoErv14 in recognizing these transmembrane proteins may be more complex.

Attenuation of appressorium formation would provide a means for managing the rice blast. Despite this extraordinary potential, however, relatively few fungicides targeting appressoria have been developed. Previously, identifying chemicals inhibiting very long-chain fatty acids (VLCFA) biosynthesis, which prevents septin-mediated appressorium formation, offers an effective strategy for controlling fungal diseases [27]. Our study could provide another avenue for controlling the disease by interfering with MoErv14-mediated ER-Golgi trafficking.

## Methods and materials

### Strains and cultural conditions

The *M*. *oryzae* strain Guy11 was used as the wild-type strain for transformation in this research. All strains were cultured on a complete medium (CM) at 28°C [28,29]. For vegetative growth, small blocks were cut from the edge of 7-day-old cultures and placed onto fresh media, followed by incubation in the dark at 28°C. The radial growth was then measured after incubation for seven days. Other media, including oatmeal agar medium and minimal medium, were prepared as described previously [27]. Liquid CM was used to prepare the vegetative mycelia for DNA and RNA extraction. For conidiation, mycelial blocks were inoculated on straw decoction and corn agar medium (SDC) (100 g of rice straw decoction was boiled in 1 L of ddH_2_O for 20 min and filtered, and the filtrate was mixed with 40 g of cornmeal and 15 g of agar and adjusted to 1 L with ddH_2_O) at 28°C for 7 d in the dark and followed by 3 days of continuous illumination under fluorescent light [30].

### Co-immunoprecipitation (co-IP) assay

The empty GFP construct was obtained in our previous work [28]. The *MoERV14* DNA fragment fused with the S tag was inserted into the pXY203 construct (*MoERV14-S*) containing the hygromycin resistance, and the *MoPTH11*, *MoWISH*, and *MoSHO1* DNA fragment fused with GFP fluorescent protein was inserted into the pYF11 construct, respectively. Then MoPth11-GFP/MoErv14-S, MoWish-GFP/MoErv14-S, MoSho1-GFP/MoErv14-S, and MoErv14-S/GFP fusion constructs were co-transformed into wild-type strain Guy11, and transformants resistant to hygromycin and bleomycin were isolated. Total proteins were extracted from the transformants using protein lysis buffer (50 mM Tris-HCl, pH 7.4, with 150 mM NaCl, 1 mM EDTA, and 1% Triton X-100 [Sigma-Aldrich, T8787]) and incubated with anti-GFP agarose for 4 h, followed by washing the affinity gel with Tris-buffered saline (TBS) (50 mM Tris-HCl, 150 mM NaCl, pH 7.4) four times. The proteins bound to the affinity gel were eluted by 0.1 M glycine HCl (pH 2.5) and were detected by anti-S and anti-GFP antibodies (Abmart, Shanghai, China) [11].

### Yeast two-hybrid assay

Full-length cDNA of MoPth11, MoWish, and MoSho1 (see primers in S1 Table) was cloned into the prey pGADT7 and the bait pGBKT7-MoErv14 constructs, respectively, and the plasmid pairs were co-transformed into the yeast strain AH109 according to the description (Clontech, USA). The transformants from SD-Trp-Leu plates were then isolated and spotted on SD-Trp-Leu-His and SD-Trp-Leu-His-Ade media for further growth testing.

### *MoERV* gene family deletion and complementation

Five Δ*Moerv* mutants were generated by using the one-step gene replacement strategy. Two fragments with 1.0 kb of sequences flanking the targeted gene were PCR amplified with primer pairs (S1 Table). The resulting PCR products were ligated to the hygromycin resistance cassette (*HPH*) released from pCX62. The 3.4-kb fragment, which included the flanking sequences and the *HPH* cassette, was transformed into Guy11 protoplasts [31]. Putative mutants were screened by PCR and further confirmed by Southern blot analysis. The complement fragment, which contains the entire *MoERV14* gene coding region and its native promoter region, was amplified by PCR with primers (S1 Table) and inserted into the pYF11 construct to complement the respective mutant strain [32].

### Conidial germination and appressorium formation

Conidial germination and appressorium formation were measured on a hydrophobic surface as described before [33–35]. Conidial suspensions of 25 μL (5×10^4^ spores/mL) were dropped onto a hydrophobic surface and placed in a humidified box at 28°C. The appressorium formation rate was counted at 24 h post inoculation (hpi) under a microscope, and more than 200 appressoria were counted for each strain.

### Host penetration and pathogenicity assays

Conidia were collected from mutants grown on SDC agar for 10 days and re-suspended by 0.2% (w/v) gelatin solution to a concentration of 5×10^4^ spores/ml. For spraying assay, two-week-old rice seedlings (*O*. *sativa cv*. CO39) were sprayed with 4 ml of the conidial suspension of each treatment and kept in a growth chamber at 25°C with 90% humidity in the dark for the first 24 h, followed by a 12-h-light and 12-h-dark cycle [36,37]. Lesion formation was daily checked and photographed after seven days of inoculation. The 7-day-old barley leaves were drop-inoculated with three droplets (20 μl) of conidial suspension, and photographs were taken 5 days after infection. Each experiment was repeated more than three times, and the experimental condition was kept consistent (e.g., temperature, humidity, illumination, and the age of the plants). For the ‘relative fungal growth’ assay, total DNA was extracted from 1.5 g disease leaves and tested by qRT-PCR (HiScript II Reverse Transcriptase, Vazyme Biotech Co., Nanjing, China) with 28S/Rubq1 primers (S1 Table) [38,39]. For rice sheath penetration and invasive hyphae expansion, conidial suspension (1×10^5^ spores/ml) was inoculated into the sheaths. After incubation for 36 h at 28°C, the sheath cuticle cells were observed under a Zeiss Axio Observer A1 inverted microscope [40].

### Organelle isolation

Protoplasts were prepared and maintained in a buffer containing 100 mM Tris-HCl, 0.1 mM MgCl_2_, 10 mM DTT, and 1.1 M sorbitol plus proteinases inhibitor mix (8215, Sigma-Aldrich), as previously described [32]. Organelles were isolated according to the established protocols with minor modifications as follows. Briefly, protoplasts were lysed in a buffer containing 10 mM Tris-HCl, 0.5 mM MgCl_2_, 8% Ficoll, and a proteinase inhibitor mixture, and the lysates were placed on the top of a centrifuge tube containing a bottom layer of the lysis buffer (10 ml) and a top layer of the same buffer containing 4% Ficoll (10 ml). Intact organelles, which are viewed as an opaque layer at the top of the centrifuge tube following centrifugation (50 000 g, 45 min), were collected. The supernatants were loaded into a cushion of 18% iodixanol (Sigma-Aldrich) for density gradient self-generation. After precipitation at 100 000 g for 2 h in a swinging bucket rotor, the crude membranes were collected at the interface, adjusted to 16% in iodixanol, and spun again at 350 000 g for 3 h. Fractions of 0.5 ml were harvested and washed with 0.8 ml 160 mM Na_2_CO_3_ for 30 min at 4°C. The membrane fraction was precipitated (100 000 g), washed with water, and then precipitated again. Membranes were solubilized in a buffer (0.1 ml of 25 mM triethylammonium bicarbonate/8 M urea/2% Triton X-100/0.1% SDS), and concentrations were determined.

### FRAP assay

Germinated conidia with 3 h of incubation were treated with cycloheximide and benomyl as described [19]. FRAP was performed using a fluorescence microscope Zeiss LSM710. Regions containing MoPth11-GFP, MoWish-GFP, and MoSho1-GFP in germ tubes were selected for photo-bleaching. Photobleaching was carried out using an Argon-multiline laser at a wavelength of 488 nm with 90% laser power and 150 iterations in ROI. Images were acquired with 2% laser power at a wavelength of 488 nm every 5 sec. For quantitative analyses, the fluorescence intensity was measured using the ZEISS ZEN blue software, and fluorescence recovery curves were fitted using the following formula: F(t) = Fmin + (Fmax—Fmin)(1-exp-kt), where F(t) is the intensity of fluorescence at time t, Fmin is the intensity of fluorescence immediately post-bleaching, Fmax is the intensity of fluorescence following complete recovery, and k is the rate constant of the exponential recovery. Mobile Fraction was calculated as the following formula: Mf = (Fend—F0)/(Fpre—F0), where Fend is the stable fluorescent intensity of the punctae after sufficient recovery, F0 is the fluorescent intensity immediately after bleaching, and Fpre is the fluorescent intensity before bleaching. The germinated conidia (3 h) with cycloheximide inhibit protein biosynthesis, which may prevent Golgi resident MoPth11-GFP, MoWish-GFP, and MoSho1-GFP from entering endosomes. The germinated conidia also treated with benomyl for 10 min to inhibit endosome trafficking.

### The extraction and purification of vesicles

About 5 g mycelia were ground in liquid nitrogen with a mortar and pestle, and the mycelia powder was suspended in 15 ml of 0.1 M sodium acetate (containing 0.07% β-mercaptoethanol), then stirred at 4°C for 2 h. The mixture was centrifuged at 6500 g for 30 min and the supernatant was transferred to a new centrifuge tube. The precipitation was resuspended in 15 ml 0.1 M sodium acetate containing 0.07% β-mercaptoethanol. After being well-suspended, the mixture was centrifuged at 6500 rpm for 30min, and the supernatant was mixed with the previous one. In order to remove the debris, the mixed supernatant was centrifuged at 7500 g for 30 min at 4°C. The supernatant was again centrifuged at 50000 g for 90 min. The supernatant was removed and the precipitate dissolved in 1 ml of TMD buffer (50 mM Tris pH 7.5, 10 mM MgCl_2_, 5 mM DTT) for 30 min. The mixture was again precipitated at 8000 g for 3 min at 4°C, and glycerol was added to a final concentration of 15% to the supernatant. The suspension was stored at -80°C.

### The lysis and extraction of vesicle proteins

For protein extraction, 400 μl vesicle samples were dissolved with 200 μl of lysis buffer (5 M urea, 2 M thiourea, 2% CHAPS, 2% SB 3–10, 40 mM Tris, 0.07% β-mercaptoethanol, 1 mM PMSF) and mixed uniformly. Proteins were precipitated by adding two volumes of pre-cold acetone containing 10% TCA and 0.07% β-mercaptoethanol and kept at -20°C for 30 min before precipitating at 13000 g, 4°C for 30 min. The pellet was washed with pre-cold acetone 3 times and air dried. The protein sample was re-dissolved in 200 μl of lysis buffer and centrifuged at 13000 g to increase impurity. Further purification was carried out using the 2D Clean-Up kit (GE Healthcare).

## Supporting information

S1 Fig Targeted genes knockout strategy and confirmation by Southern blot analysis. (A-E) The strategy of knocking out target genes in *M*. *oryzae* genome. Thin lines below the arrows indicate the probe sequence of each gene. Southern blot analysis was used to confirm the *MoERV14* deletion and the copy of the *HPH* gene.
(TIF)

S2 Fig MoErv14 is homolog of ScErv14. *MoERV14* could partially suppress the growth defect of the yeast Δ*Scerv14* mutant under 30 μg/ml DTT stress. The yeast Δ*ScERV14* mutant was complemented with *MoERV14* cDNA. The yeast wild type-strain BY4741 and the Δ*Scerv14* mutant transformed with the empty pYES2 vector were used as controls. Serial dilutions of cell suspensions of each strain were spotted on SD and SD+DTT plates for 5 days and photographed.
(TIF)

S3 Fig MoErv14 is involved in appressorium turgor generation. Statistical analysis of the collapsed appressoria on hydrophobic surfaces after 24 h incubation. Error bars represent ±SD, and asterisks represent significant differences (*p < 0*.*01*).
(TIF)

S4 Fig MoErv14 is dispensable for septin-actin assembly. Septin network in appressoria (24 h) of Guy11 and Δ*Moerv14* mutant.Bars, 5 μm.
(TIF)

S5 Fig MoErv14 is a vesicle protein and located at ER and Golgi. (A and B) The localization pattern of MoErv14 in conidia and appressorium phase. Left, the observation of MoErv14-GFP with ER marker MoLsh1. Right, the observation of MoErv14-GFP with Golgi marker MoSft2. Bars, 10 μm (C) Organelles from *M*. *oryzae* protoplasts were partially separated by centrifugation. The ER and Golgi distribution were analyzed by Western blotting, using RFP antibodies against the ER marker MoLhs1 and Golgi marker MoSft2 fused with RFP. Distribution of MoErv14 was detected by anti-s antibody. (D) The extraction of the vesicular proteins from MoErv14-GFP/MoSec24-2-RFP co-transformed strains and used the western blot to detect MoErv14 and MoSec24-2-RFP. The MoSec24-2-RFP and anti-tubulin were used as references.
(TIF)

S6 Fig MoErv14 is dispensable for endocytosis. Hyphae stained by FM4-64 were examined by using fluorescence microscopy at different time points to observe the FM4-64 uptake. Bars, 10 μm.
(TIF)

S7 Fig MoErv14 is important for intracellular cAMP generation and MoPmk1 phosphorylation. (A) Loss of *MoERV14* leads to decreased accumulation of cAMP. Bar chart showing quantification of intracellular cAMP in the mycelia of the indicated strains cultured for 2 days in complete medium. Two biological repetitions with three replicates were assayed. The error bars represent SD of three replicates. The asterisks denote statistical significances (*p*<0.01). (B) The total protein of Guy11 and *ΔMoerv14* mutant strains were isolated from mycelia for detecting the MoPmk1 phosphorylation level using the anti-phospho-p44/42 MAP kinase antibody (Cell Signaling Technology) and the anti-p44/42 MAP kinase antibody (Cell Signaling Technology) was used as control. Three independent experiments were replicated that showed similar results. The asterisks denote statistical significance (*p*<0.01).
(TIF)

S8 Fig There is no interaction detected among MoPth11, MoWish, and MoSho1. (A-C) Yeast two-hybrid assay for interactions among MoPth11, MoWish, and MoSho1.
(TIF)

S9 Fig MoPth11, MoWish and MoSho1 are detained in ER with BFA treatment. Three cell membrane proteins fused with GFP, then the transformats were treated with BFA and co-localized with MoLhs1-RFP. Images were observed by Axio Observer A1 Zeiss inverted microscope. Bar = 10 μm.
(TIF)

S1 Table Primers used in this study. This work was supported by the Natural Science Foundation of China-German Research Foundation Mobility Programme (31861133017 to ZZG), and the China National Funds for Innovative Research Groups (Grant No.31721004 to ZZG), NSFC (31772110 to ZZG). QB received support from Natural Science Foundation of China Youth Programme (NSFC 32202240), and grant number BK20200543 fromYouth Program for Natural Science Foundation of Jiangsu Province. WP received support from grant number AI156254 and AI168867 of the National Institutes of Health (USA). The funders had no role in study design, data collection and analysis, decision to publish, or preparation of the manuscript.
(DOCX)
