# Supplementary figures and images for: MoErv14 mediates the intracellular transport of cell membrane receptors to govern the appressorial formation and pathogenicity of Magnaporthe oryzae
Source: PLoS Pathog. 2023 Apr 3;19(4):e1011251. doi: 10.1371/journal.ppat.1011251 (PMC10101639; doi:10.1371/journal.ppat.1011251)

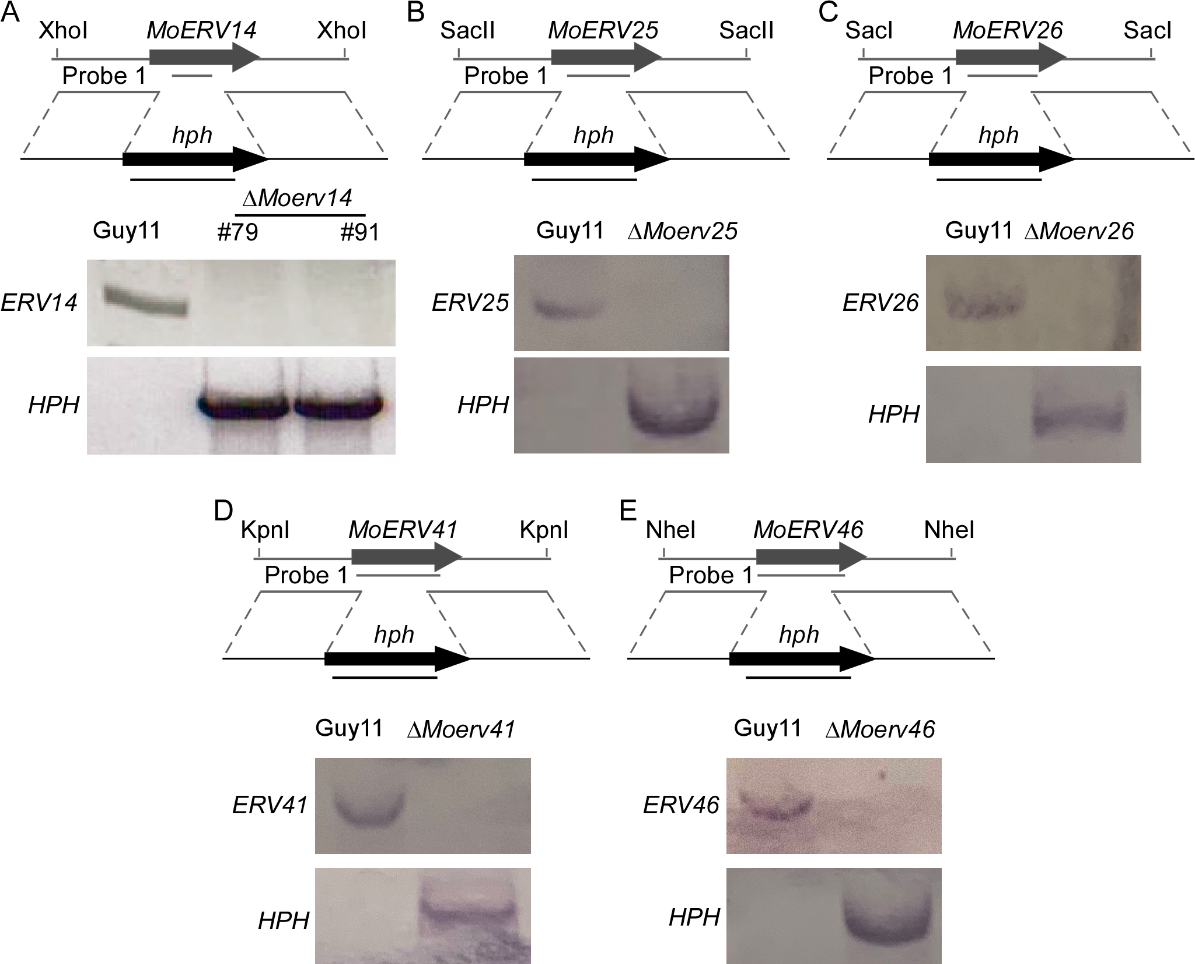

Supplement: S1 Fig — (A-E) The strategy of knocking out target genes in M. oryzae genome. Thin lines below the arrows indicate the probe sequence of each gene. Southern blot analysis was used to confirm the MoERV14 deletion and the copy of the HPH gene. (TIF) [file ppat.1011251.s001.tif]

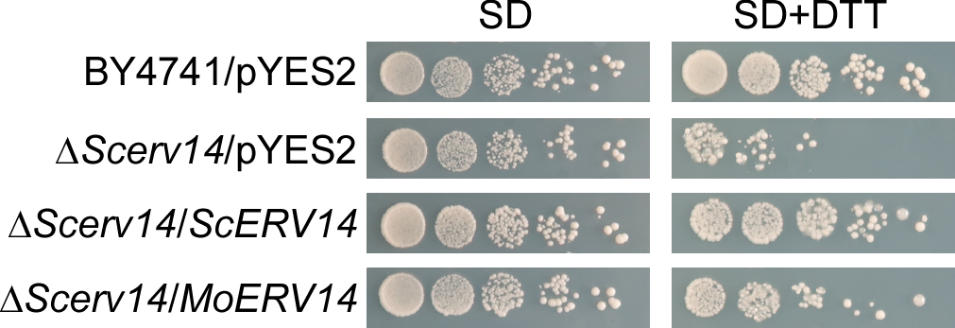

Supplement: S2 Fig — MoERV14 could partially suppress the growth defect of the yeast ΔScerv14 mutant under 30 μg/ml DTT stress. The yeast ΔScERV14 mutant was complemented with MoERV14 cDNA. The yeast wild type-strain BY4741 and the ΔScerv14 mutant transformed with the empty pYES2 vector were used as controls. Serial dilutions of cell suspensions of each strain were spotted on SD and SD+DTT plates for 5 days and photographed. (TIF) [file ppat.1011251.s002.tif]

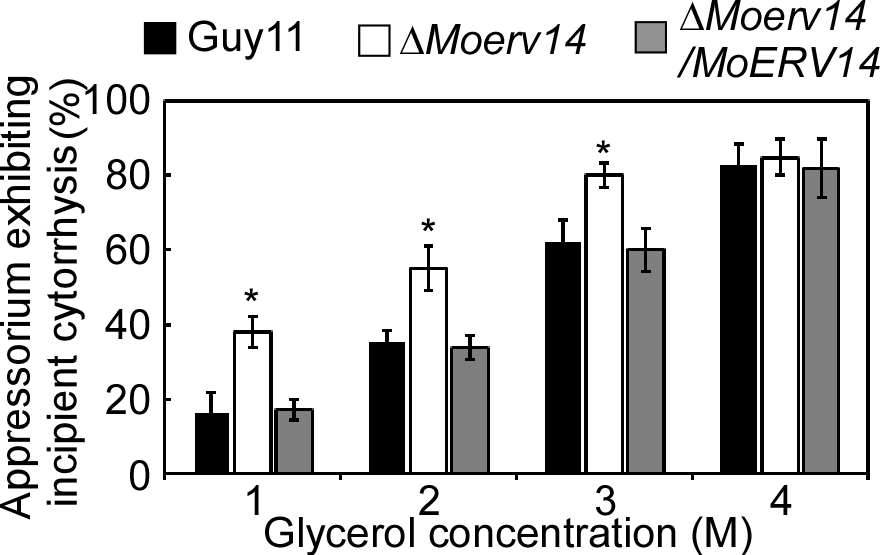

Supplement: S3 Fig — Statistical analysis of the collapsed appressoria on hydrophobic surfaces after 24 h incubation. Error bars represent ±SD, and asterisks represent significant differences (p < 0.01). (TIF) [file ppat.1011251.s003.tif]

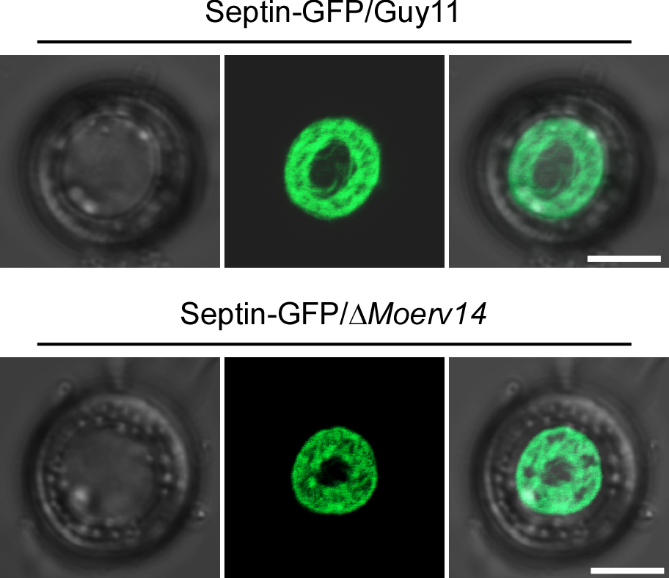

Supplement: S4 Fig — Septin network in appressoria (24 h) of Guy11 and ΔMoerv14 mutant.Bars, 5 μm. (TIF) [file ppat.1011251.s004.tif]

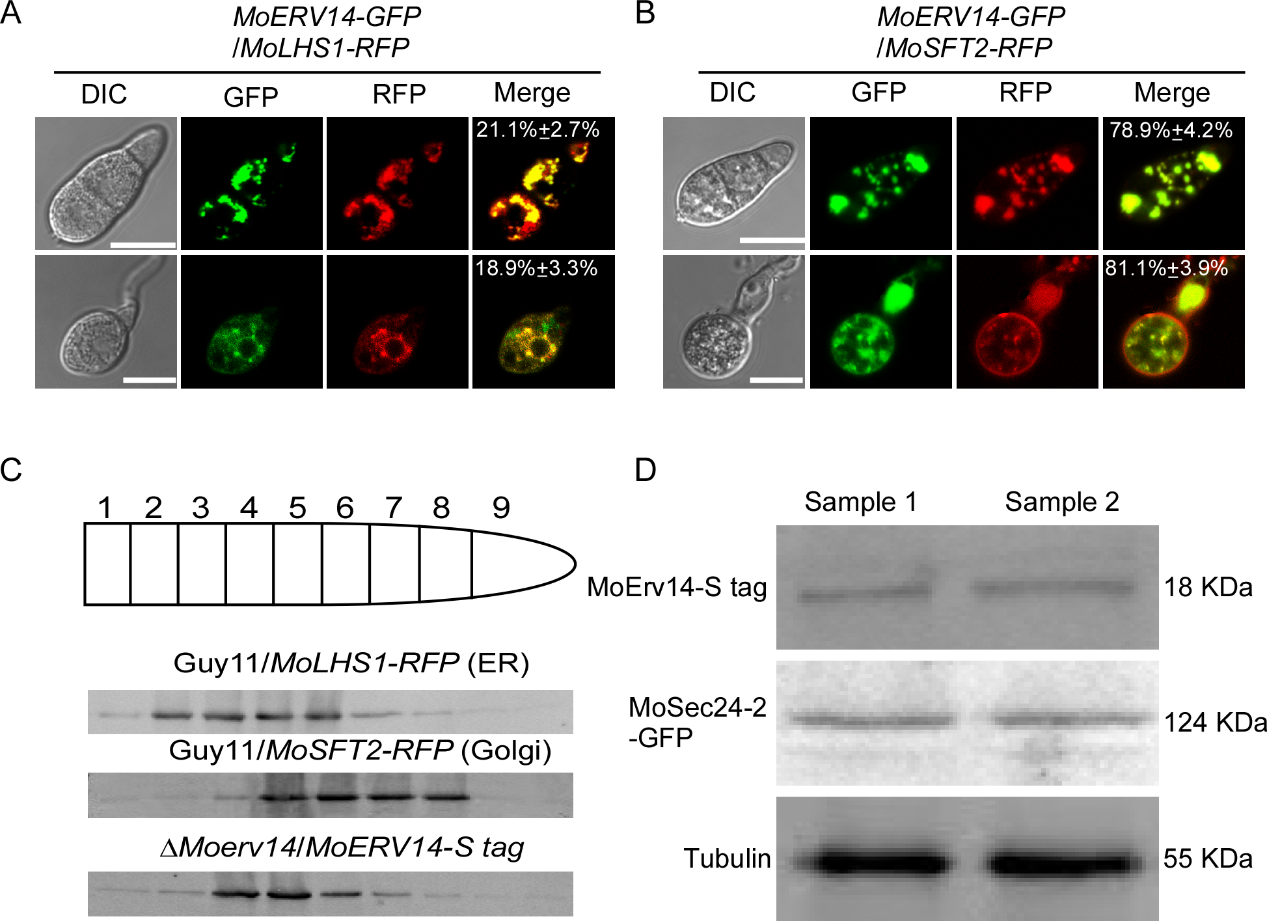

Supplement: S5 Fig — (A and B) The localization pattern of MoErv14 in conidia and appressorium phase. Left, the observation of MoErv14-GFP with ER marker MoLsh1. Right, the observation of MoErv14-GFP with Golgi marker MoSft2. Bars, 10 μm (C) Organelles from M. oryzae protoplasts were partially separated by centrifugation. The ER and Golgi distribution were analyzed by Western blotting, using RFP antibodies against the ER marker MoLhs1 and Golgi marker MoSft2 fused with RFP. Distribution of MoErv14 was detected by anti-s antibody. (D) The extraction of the vesicular proteins from MoErv14-GFP/MoSec24-2-RFP co-transformed strains and used the western blot to detect MoErv14 and MoSec24-2-RFP. The MoSec24-2-RFP and anti-tubulin were used as references. (TIF) [file ppat.1011251.s005.tif]

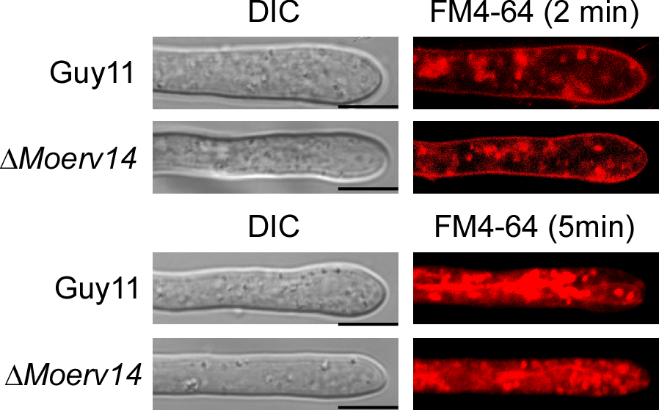

Supplement: S6 Fig — Hyphae stained by FM4-64 were examined by using fluorescence microscopy at different time points to observe the FM4-64 uptake. Bars, 10 μm. (TIF) [file ppat.1011251.s006.tif]

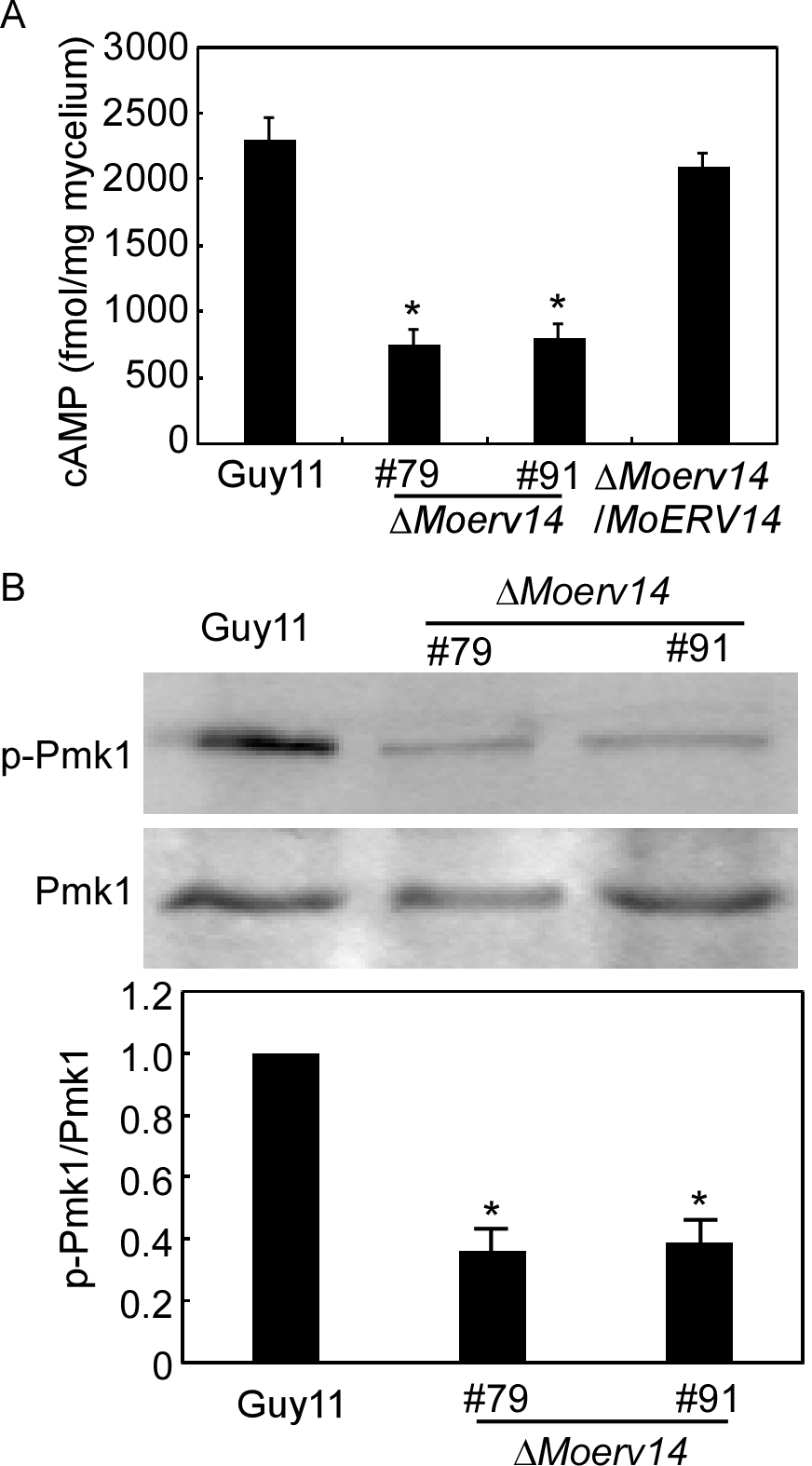

Supplement: S7 Fig — (A) Loss of MoERV14 leads to decreased accumulation of cAMP. Bar chart showing quantification of intracellular cAMP in the mycelia of the indicated strains cultured for 2 days in complete medium. Two biological repetitions with three replicates were assayed. The error bars represent SD of three replicates. The asterisks denote statistical significances (p<0.01). (B) The total protein of Guy11 and ΔMoerv14 mutant strains were isolated from mycelia for detecting the MoPmk1 phosphorylation level using the anti-phospho-p44/42 MAP kinase antibody (Cell Signaling Technology) and the anti-p44/42 MAP kinase antibody (Cell Signaling Technology) was used as control. Three independent experiments were replicated that showed similar results. The asterisks denote statistical significance (p<0.01). (TIF) [file ppat.1011251.s007.tif]

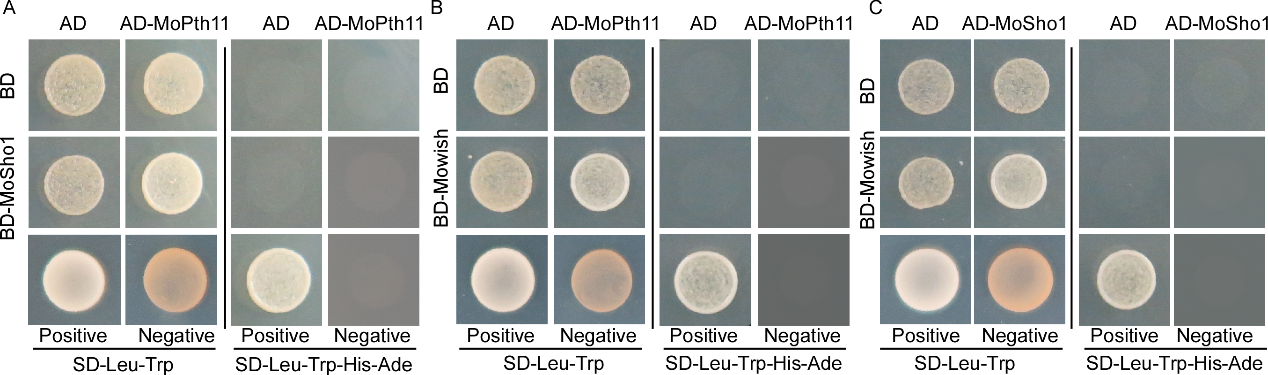

Supplement: S8 Fig — (A-C) Yeast two-hybrid assay for interactions among MoPth11, MoWish, and MoSho1. (TIF) [file ppat.1011251.s008.tif]

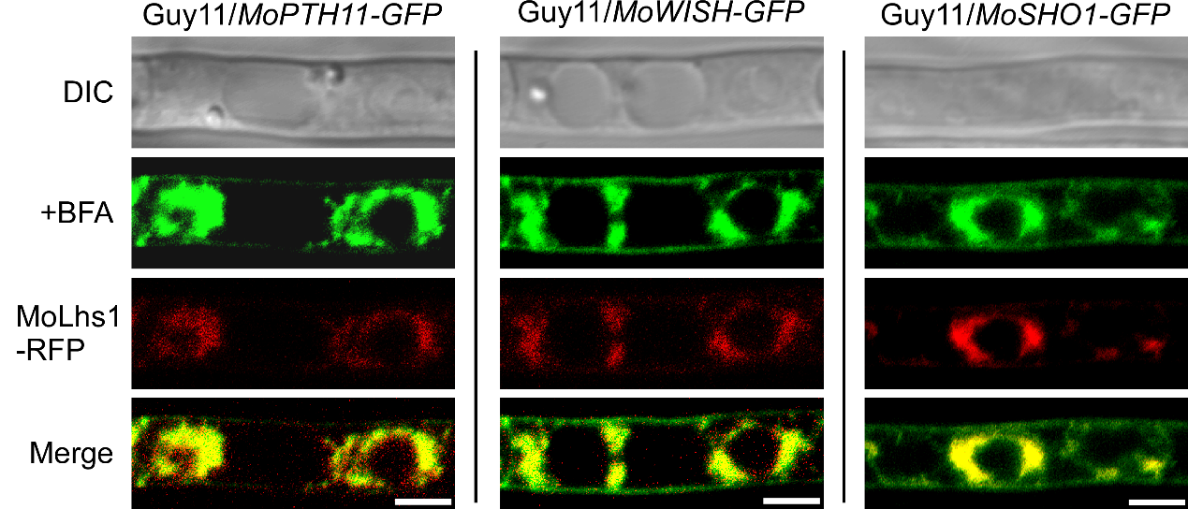

Supplement: S9 Fig — Three cell membrane proteins fused with GFP, then the transformats were treated with BFA and co-localized with MoLhs1-RFP. Images were observed by Axio Observer A1 Zeiss inverted microscope. Bar = 10 μm. (TIF) [file ppat.1011251.s009.tif]
