# Supplementary material for: MoErv14 mediates the intracellular transport of cell membrane receptors to govern the appressorial formation and pathogenicity of Magnaporthe oryzae
Source: PLoS Pathog. 2023 Apr 3;19(4):e1011251. doi: 10.1371/journal.ppat.1011251 (PMC10101639; doi:10.1371/journal.ppat.1011251)
Supplement: S1 Table — This work was supported by the Natural Science Foundation of China-German Research Foundation Mobility Programme (31861133017 to ZZG), and the China National Funds for Innovative Research Groups (Grant No.31721004 to ZZG), NSFC (31772110 to ZZG). QB received support from Natural Science Foundation of China Youth Programme (NSFC 32202240), and grant number BK20200543 fromYouth Program for Natural Science Foundation of Jiangsu Province. WP received support from grant number AI156254 and AI168867 of the National Institutes of Health (USA). The funders had no role in study design, data collection and analysis, decision to publish, or preparation of the manuscript. (DOCX) [file ppat.1011251.s010.docx]

**S1 Table. Primers used in this study**

| Primer name | Sequence (5’-3’) | Remark |
| --- | --- | --- |
| ERV14 F1 | TAAGTCGACGGATGGGGCAGAAGACAATCCAG | amplify *MoERV14* 5’ flank sequence |
| ERV14 R1 | TAAGATATCGTTGTGTGCGTGCGATGGCTTG | amplify *MoERV14* 5’ flank sequence |
| ERV14 F2 | TAAGGATCCCTGTGCGGAGTTCTTGTCCATC | amplify *MoERV14* 3’ flank sequence |
| ERV14 R2 | TAAGAGCTCGAAAACTGGGTCCTGAAGGTG | amplify *MoERV14* 3’ flank sequence |
| ERV14 KO-F | CACCTTTTGCGCCGTTATCTC | amplify *MoERV14* probe sequence |
| ERV14 KO-R | GAACATGATCAAGTGGAAGC | amplify *MoERV14* probe sequence |
| ERV14 BY | CAACTACCATGCGACTACCAC | validation of *MoERV14* deletion (HPH) |
| HPH R | GCTGATCTGACCAGTTGCCTA | validation of *MoERV14* deletion (HPH) |
| ERV14 HB-F1 | ACTCACTATAGGGCGAATTGGGTACTCAAATTGGTTCAACTACCATGCGACTACCAC | *MoERV14* complementation |
| ERV14 HB-R1 | CACCACCCCGGTGAACAGCTCCTCGCCCTTGTGTGACTCGTCCTTGATC | *MoERV14* complementation |
| AD-ERV14 F1 | TACCAGATTACGCTCATATGATGTCTGGGGAAGCATGGTTATACC | Construction of AD-ERV14 |
| AD-ERV14 R1 | TGCCCACCCGGGTGGAATTCTTAGTGTGACTCGTCCTTGATC | Construction of AD-ERV14 |
| BD-ERV14 F1 | TGATCTCAGAGGAGGACCTGCATATGATGTCTGGGGAAGCATGGTTATACC | Construction of BD-ERV14 |
| BD-ERV14 R1 | GCAGGTCGACGGATCCCCGGGAATTCTTAGTGTGACTCGTCCTTGATC | Construction of BD-ERV14 |
| Erv14 Stag F1: | TTTCGTAGGAACCCAATCTTCAAAATGTCTGGGGAAGCATGGTTATAC | Construction of Erv14-S |
| Erv14 StagR1: | TTCGAATTTAGCAGCAGCGGTTTCTTTGTGTGACTCGTCCTTGATC | Construction of Erv14-S |
| ERV25 F1 | TAACTCGAGGAACGCCTCCGGGGAAGCCAAATG | amplify *MoERV25* 5’ flank sequence |
| ERV25 R1 | TAAGAATTCTTTGTTGGTCCTGCTGGCTG | amplify *MoERV25* 5’ flank sequence |
| ERV25 F2 | TAAACTAGTCGCAAAAATAAGACATGCTGTGC | amplify *MoERV25* 3’ flank sequence |
| ERV25 R2 | TAAGAGCTCCTACTTTTTGCTCCTCGATCTGC | amplify *MoERV25* 3’ flank sequence |
| ERV26 F1 | TAACTCGAGGCGTACAGGCCGTTGAAGAAC | amplify *MoERV26* 5’ flank sequence |
| ERV26 R1 | TAAGAATTCCTTGGGCGAATGATCTGATGG | amplify *MoERV26* 5’ flank sequence |
| ERV26 F2 | TAAACTAGT TGACTTATGGGTGTTTATCG | amplify *MoERV26* 3’ flank sequence |
| ERV26 R2 | TAACCGCGGTTATCTTACCACCCAGCGGC | amplify *MoERV26* 3’ flank sequence |
| ERV41 F1 | TAACTCGAGAGATGTTGCTGAGGGATAAG | amplify *MoERV41* 5’ flank sequence |
| ERV41 R1: | TAAGAATTCTGTGCCGGCGGGTTTGGGGAG | amplify *MoERV41* 5’ flank sequence |
| ERV41 F2  ERV41 R2 | TAAACTAGT AAGGGTGTGTCTGCATCTG  TAACCGCGGGACAACCAAGATTTTGCTAC | amplify *MoERV41* 3’ flank sequence  amplify *MoERV41* 3’ flank sequence |
| Erv46 F1: | TAAGGTACCGAGAAGATGATGCTGCTGTTG | amplify *MoERV46* 5’ flank sequence |
| Erv46 R1: | TAAGAATTCGTGTGGGTTGGCTTGTTCCTC | amplify *MoERV46* 5’ flank sequence |
| Erv46 F2: | TAAACTAGTCCACGAGACTCATAAAAAATCC | amplify *MoERV46* 5’ flank sequence |
| Erv46 R2: | TAACCGCGGGACACCCGCTTTTGTTGCGAG | amplify *MoERV46* 5’ flank sequence |
| MoERV25 BY: | CGATGGCGAGGTTAGCGTTTGCTG | validation of *MoERV25* deletion (HPH) |
| MoERV25 ConF: | CCTCATGCAGATCAAGGATGC | amplify *MoERV25* probe sequence |
| MoERV25 ConR: | GCACGCAGGTACATAATCTGC | amplify *MoERV25* probe sequence |
| ERV26 ConF: | GAGCTTGTCGAGGAGCACACGGTC | amplify *MoERV26* probe sequence |
| ERV26 ConR | CGTTGTCGCTAGCTGACAGGCTTATG | amplify *MoERV26* probe sequence |
| ERV26 BY: | CTGACAGAATGGGTTACAGAG | validation of *MoERV26* deletion (HPH) |
| ERV41 ConF: | CTCAAGATGGACGACACCACCTG | amplify *MoERV41* probe sequence |
| ERV41ConR: | CTCGCTGATCTCGGAGCTCTGCTC | amplify *MoERV41*probe sequence |
| ERV41 BY： | CTATCTACTGCTGGGACTGCAG | validation of *MoERV41* deletion (HPH) |
| MoErv46 ConF: | CGAGCAACAACACGGCGTGCAGCAC | amplify *MoERV46* probe sequence |
| MoErv46 ConR | CATGTTCTTGTCCTTGTTGCCAAGC | amplify *MoERV46* probe sequence |
| MoErv46 BY: | GAGCCCATCTTGAACAACAG | validation of *MoERV46* deletion (HPH) |
| AD-Wish F1 | TACCAGATTACGCTCATATGATGAAGCTGTTGCACTTTTTCC | Construction of AD-Wish |
| AD-Wish R1 | TGCCCACCCGGGTGGAATTCTTA TTTCCGGTTCGCATTGGTAG | Construction of AD-Wish |
| AD-Pth11 F1 | TACCAGATTACGCTCATATGATGGTTGCATTCACCCGGTTGCTGCTTG | Construction of AD-Pth11 |
| AD-Pth11 R1 | TGCCCACCCGGGTGGAATTCTTATTAGATGAGACCACCGGGCAG | Construction of AD-Pth11 |
| AD-Sho1 F1 | TACCAGATTACGCTCATATGATGCCATCATACGGCTCGCTGCATTC | Construction of AD-Sho1 |
| AD-Sho1 R1  28S rDNA LL  28S rDNA RR  Rubq1 LL  Rubq1 RR | TGCCCACCCGGGTGGAATTC TCATAGCAAAATAAGGTAGTTAC  TACGAGAGGAACCGCTCATTCAGATAATTA  TCAGCAGATCGTAACGATAAAGCTACTC  GTGGTGGCCAGTAAGTCCTC  GGACACAATGATTAGGGATCA | Construction of AD-Sho1  qPCR  qPCR  qPCR  qPCR |
|  |  |  |
